# Tuakana-teina peer education programme to help Māori elders enhance wellbeing and social connectedness

**DOI:** 10.1186/s12877-024-04703-0

**Published:** 2024-01-30

**Authors:** John G. Oetzel, Mary Simpson, Pare Meha, Michael P. Cameron, Yingsha Zhang, Sophie Nock, Rangimahora Reddy, Hariata Adams, Ngapera Akapita, Ngareo Akariri, Justina Anderson, Marama Clark, Kawarau Ngaia, Brendan Hokowhitu

**Affiliations:** 1 University of Waikato, Private Bag 3105, 3240 Hamilton, New Zealand; 2 Rauawaawa Kaumātua Charitable Trust, 50 Colombo St, 3204 Hamilton, New Zealand; 3 Sun Yat-sen University, Zhuahi, Guangdong China; 4 Te Korowai Hauora o Hauraki, 210 Richmond St, 3500 Thames, New Zealand; 5 Ngāti Ruanui Whānau Ora, 96 Collins Street, 4610 Hawera, New Zealand; 6 Tui Ora Limited, 36 Maratahu Street, 4342 New Plymouth, New Zealand; 7 Poutiri Trust, 35 Commerce Lane, 3119 Te Puke, New Zealand; 8 Te Korowai o Ngāruahine Trust, 4610 Hawera, PO Box 474, New Zealand; 9 University of Queensland, Brisbane, Australia

**Keywords:** Kaupapa Māori, tuakana-teina, Peer education, Positive ageing, Community-based participatory research, Mana Motuhake

## Abstract

**Background:**

There are significant inequities between Māori (Indigenous people) and non-Māori in ageing outcomes. This study used a strengths-based approach based on the key cultural concept of mana motuhake (autonomy and self-actualisation) to develop a tuakana-teina (literally older sibling-younger sibling) peer education programme to assist kaumātua (elders) in addressing health and social needs. The purpose of this study was to test the impact on those receiving the programme. Three aims identify the impact on outcomes, resources received and the cost effectiveness of the programme.

**Methods:**

Five Kaupapa Māori (research and services guided by Māori worldviews) iwi (tribe) and community providers implemented the project using a partnership approach. Tuakana (peer educators) had up to six conversations each with up to six teina (peer learners) and shared information related to social and health services. A pre- and post-test, clustered staggered design was the research design. Participants completed a baseline and post-programme assessment of health and mana motuhake measures consistent with Māori worldviews. Open-ended questions on the assessments, five focus groups, and four individual interviews were used for qualitative evaluation.

**Findings:**

A total of 113 kaumātua were recruited, and 86 completed the programme. The analysis revealed improvements in health-related quality of life, needing more help with daily tasks, life satisfaction, paying bills and housing problems. Qualitative results supported impacts of the programme on mana motuhake and hauora (holistic health) through providing intangible and tangible resources. Cost-effectiveness analysis showed that the intervention is cost effective, with a cost per QALY of less than the conventional threshold of three times GDP per capita.

**Conclusions:**

A culturally-resonant, strengths-based programme developed through a participatory approach can significantly improve health and social outcomes in a cost-effective way.

**Trial Registry:**

**Clinical trial registry**: Trial registration: (ACTRN12620000316909). *Prospectively registered* 06/03/2020, https://www.anzctr.org.au/Trial/Registration/TrialReview.aspx?id=379302&isClinicalTrial=False.

## Background

Kaumātua (older Māori; Indigenous people) in Aotearoa New Zealand experience significant health inequities compared to non-Māori elders [1–3]. Russell et al. [4] highlighted that “Māori experience systematic disparities in health outcomes, determinants of health, health system responsiveness, and representation in the health sector workforce” (p.10). These inequities result from lack of access to health and/or social services, the unequal distribution of social determinants (e.g., housing, education, income), and structural discrimination resulting from the effects of colonisation [1, 3, 5].

These inequities and patterns are consistent with other Indigenous peoples, cultures and communities worldwide [6, 7]. “Globally, health disparities between Indigenous and non-Indigenous populations are ubiquitous and pervasive, and are recognized as being unfair, avoidable, and remediable (p. 512)” [8]. Thus, the deleterious impacts of colonisation on the Indigenous life-course are endemic internationally. Further, the fact that Indigenous peoples die earlier than their non-Indigenous compatriots creates a great pain and a sense of loss for Indigenous cultures that view their elders as bearers of knowledge critical to survivance [9]. Māori culture upholds kaumātua as, “carriers of culture, anchors for families, models for lifestyle, bridges to the future, guardians of heritage, and role models for younger generations (p. 14)” [10].

Māori make up about 17% of the total population in Aotearoa and about 7% of Māori are aged 65 years and older [11]. Growing up in the 1940-60s, many of today’s kaumātua experienced a more racist society than present, including government policies that fostered monoculturalism and an education system that discouraged and punished children for speaking Te Reo Māori (Māori language) and/or for practicing tikanga Māori (Māori cultural protocols) [12]. Currently, there are many kaupapa Māori health and social service providers (providers that operate using a Māori worldview) that are funded to address many of the negative health effects arising from this history. However, these providers are typically underfunded, and inequities persist. In the past two years, the New Zealand health system has been reorganised to address these inequities including the development of Te Aka Whai Ora (the Māori Health Authority) and increased funding to support Māori providers.

Because of this historical context and decades of insufficient funding, there are relatively few examples of innovative research programmes to address the health and social needs of kaumātua. However, recent research has developed novel health programmes (i.e., interventions) aimed at addressing such needs [13, 14]. Even so, the benefits of initiatives from Māori health providers, and other Indigenous evidence, are rarely reported in the literature [15]. Thus, communities and providers who may benefit from awareness of these programmes and implementing the initiatives, may miss out on key opportunities to address health inequities.

An exception to this pattern in the literature is the tuakana-teina peer education programme. The tuakana-teina (literally, older sibling-younger sibling, but more specifically in this programme—with and without experience) programme is where kaumātua work with other kaumātua to help them work through life transitions and identify health and social services that they might need to help address these transitions [16–18]. The programme uses a strengths-based approach that highlights the potential of kaumātua to be solutions to their own challenges, building on the strength of their mana (status) within Māori culture [17]. This strengths-based approach was based in kaumātua mana motuhake; mana motuhake emphasises autonomy and independence to achieve actualisation so that kaumātua can enhance wellbeing and quality of life for themselves and others [19]. This strengths-based approach contrasts with the predominant deficit models for health inequities that focus on dependency and weakness [20].

The tuakana-teina model has been widely used in areas such as governance, environmental resources, and school mentoring [21, 22]. This specific tuakana-teina programme was co-developed through a participatory research approach by a Māori community organisation and a group of university researchers and based on an original idea offered by kaumātua [17, 23]. This programme departs from the traditional tuakana-teina model in that the relationships were not based in genealogy and age although tuakana and teina were matched on sex; the relationships were based on experiences and skills. We have discussed these departures elsewhere; briefly we went through an in-depth co-design process with a kaumātua advisory board and a health and social services expert advisory board in determining the nature of the relationships and name for the programme [17]. These boards considered other possible labels but decided that tuakana-teina was the most appropriate description despite these departures from the traditional use of the model.

The tuakana-teina programme is also grounded in the extant literature on peer education, including the theoretical (e.g., social learning theory [24], theory of reasoned action [25], diffusion of innovation theory [26]) and empirical literature, which together demonstrates that peer support/education is effective for improving numerous economic, social and health conditions [27–30]. Peer education is offered by non-professionals to people who are of similar characteristics (e.g., culture, health, age) experiencing a social or health issue [31]. Peer education and support creates new relationships that are distinct from existing family, community and organisational relationships [31].

Earlier research has found that the tuakana-teina programme enhanced tuakana (experienced peers) communication skills to enact their peer educator role and this impacted positively on their sense of cultural identity, sense of purpose, and wellbeing [18]. The tuakana-teina programme also increased teina (inexperienced peers) perceived support, knowledge of cultural protocols, social connectedness, and informational support about health and social services [32]. Additionally, the programme was found to be cost-effective in addressing key social and health outcomes [32].

The current study builds on the original tuakana-teina programme and addresses several limitations. First, the original study only included a single community provider; we extended the current research to one iwi (tribe) and four community providers. Second, the original tuakana-teina programme only included three conversations, which may not have been enough to establish a strong relationship between the tuakana and teina; the current programme involves six conversations. Finally, the original tuakana-teina programme primarily included kaumātua with strong social and cultural resources. The current study included more participants with key health and social service needs.

Similar to the original programme, the current study focused on two broad outcomes of peer education: hauora (holistic health) and mana motuhake. Māori models of health include multiple elements of hauora (health and wellbeing): hinengaro (mental wellbeing), kare ā-roto (emotional wellbeing), whanaungatanga (social wellbeing), wairua (spiritual wellbeing), tinana (physical well-being) and tikanga (cultural wellbeing). This holistic perspective of health reflects Māori views of the relationship of people to all aspects in the world [33]. Mana motuhake is indicated by such elements as economic wellbeing, life satisfaction and personal autonomy [19].

This project sought to answer a single broad research question: What are the outcomes of the tuakana-teina programme for kaumātua with the greatest health and social needs? This study serves several specific aims: (a) To determine whether the tuakana-teina programme enhanced the social and health outcomes (hauora and mana motuhake); (b) To identify the types of resources provided; and (c) To determine the cost-effectiveness of the programme.

## Methods

We published the study protocol for this project elsewhere and full details of the project can be found there [34]. We present here a summary of the methodology, programme, study design, sample and procedures, measures, and data analysis.

### Methodology

This project is guided by the He Pikinga Waiora (Enhancing Wellbeing) Implementation Framework [35]. This framework centres Kaupapa Māori [36, 37], a culturally resonant research methodology that normalises Māori tikanga and worldviews and emphasises Māori epistemology. The framework also includes community engagement, systems thinking, culture centredness and integrated knowledge translation. The framework is a participatory approach that utilises the unique strengths of partners, along with seeking ways to partner with end users to create a sustainable programme that is integrated within the larger health and social system [38]. The research team involves a partnership of six Māori health and social service providers with university researchers. Each provider had one or two community researchers as part of the partnership (funded to 0.50 full-time equivalent). We included two advisory groups to help support our methodology: (1) a Board Advisory Group comprised of the trustees of the original provider, to ensure that the project was kaumātua-led; and (2) an Expert Advisory Group consisting of experts in social and health issues from the aged-care sector. The boards provided stewardship for the project regarding programme development, programme content and all research methods. The project is registered with the Australia New Zealand Clinical Trial Registry (ACTRN126220000316909).

### Programme

We developed the tuakana-teina programme through a participatory process for addressing the health and social needs of kaumātua [17]. We used the label ‘programme’ rather than ‘intervention’ to be consistent with the strengths-based approach. The five providers worked with the original partnership to adapt elements of the tuakana-teina programme to fit their local community, and this process is described in detail elsewhere [39], as are details of the original programme [17].

Each provider selected three or four tuakana (one provider had three, with the remaining having four) based on effective peer educator attributes for older people [40] and attributes within Te Ao Māori (Māori worldview) such as aroha (love, compassion) pono (honesty), mana (status), and tika (fairness) [10, 12]. Orientation activities for tuakana were delivered by members of the research team, including local community researchers. The orientation included the following elements: (a) identifying important skills of tuakana-teina peer support; (b) mātāpono (principles) and uara (values); (c) types of social support; (d) Māori forms of communication; (e) communication strategies for the tuakana in their conversations with teina; and (f) a resource kete (basket) consisting of social and health services. The programme comprised of four 4-hour sessions. Booster sessions were delivered by community researchers or the university team for specific tuakana if needed.

The teina were matched to a tuakana of the same sex by a community researcher from the local provider who knew the kaumātua well. Each teina was to have six conversations with his/her tuakana over a roughly six-month period. Each tuakana was originally assigned six teina. The conversations were audio-recorded to complete a fidelity check of tuakana communication.

### Study design, procedures, and sample

The research design for the evaluation of the intervention was a pre- and post-test, staggered design with two groups (G1 and G2). The groups were clustered by provider, which were randomly selected (three providers to G1 and two to G2). G1 participated in the orientation programme initially, while G2 participated in a subsequent orientation approximately three-six months later. There were three data collections points for all participants with variations for the two groups: (a) G1: baseline, post-conversations, and then two months after the second evaluation; (b) G2: baseline, approximately two months after initial survey (but before their programme), and post conversations. Participants completed the survey via a paper-pencil form and could have a support person present. The staggered research design was chosen because it is consistent with Kaupapa Māori principles, in which withholding a programme from participants is not ethical. This ensured that all participants would be able to participate in the programme and that we were also able to provide adequate support for the administration of the programme given that the partners implemented at different times. This research design also allows for comparison and is pragmatic for interventions in the health service sector [41]. Research procedures were approved by the University of Waikato Human Research Ethics Committee (Health2019#81).

After the initial recruitment, there were a total of 12 tuakana in G1 and 7 in G2. Providers were asked to offer a list of eligible kaumātua for recruitment, based on greatest health and social needs, and then the list was randomised with a target of recruiting 24 teina at each provider. One of the G2 providers could not create such a list because of privacy issues and instead recruited participants at community meetings through a non-random method. The response rate for participants was 60% (61% in G1 and 58% in G2). Reasons for refusal included: unable to contact (*n* = 20), decline to participate (*n* = 17), and other reasons including illness or work (*n* = 22).

### Measures

There were two core constructs for this study along with demographic items: *hauora* and *mana motuhake*. Quantitative measures are detailed elsewhere [34] and were validated in a separate study [16]. Table 1 lists the core constructs, measures/items, number of items and Cronbach’s alpha calculated for this study. In addition, an open-ended question was included on the survey asking participants to describe what they thought of the programme and any recommendations they had for it.

**Table 1:** Constructs and measures

Construct	Measure/Item [source]	Number of Items	Cronbach’s Alpha
Hauora-mental and physical wellbeing	Health-related quality of life (HRQOL) [42]	7	0.88
Hauroa-spiritual wellbeing	Spirituality [43]	1	n/a
Hauora-health services	Knowledge and use of using services [23]	3	0.64
Hauora-social wellbeing	Loneliness [44]	1	n/a
Hauora-social wellbeing	Needing more help with daily tasks [45]	1	n/a
Hauora-social wellbeing	Needing more help with emotional support [45]	1	n/a
Hauora-cultural wellbeing	Cultural/tribal identity [46]	2	0.71
Hauora-cultural wellbeing	Knowledge of tikanga [46]	1	n/a
Mana motuhake-Autonomy	Perceived autonomy [47]	1	n/a
Mana motuhake-safety	Elder abuse [34]	4	0.71
Mana motuhake-general satisfaction	Life satisfaction [48]	1	n/a
Mana motuhake-economic wellbeing	Trouble paying bills [43]	1	n/a
Mana motuhake—economic wellbeing	Housing problems [43]	3	0.65

Following our study protocol [34] and previous evaluation approaches [32], we also conducted five focus groups and four individual interviews with teina post-intervention (three in G1 and two in G2). There were 42 participants (75% wāhine [women]). Questions examined participants’ experiences, impact of the programme on them and their whānau (extended family), and recommendations for the programme in the future. A community researcher from the providers conducted the focus groups. Except for one provider, the focus groups were audio-recorded and professionally transcribed. This other group was unable to make a quality recording of the group and thus took detailed notes. The individual interviews were completed via written response format. This approach was chosen by one provider due to COVID pandemic restrictions.

### Data Analysis

The first specific aim sought to determine whether the peer-educator model results in changes to hauora and mana motukahe. The analysis involved multilevel analysis of mixed models using SPSS 28.0 following procedures to isolate the effect of the intervention across different groups at different times using average treatment effect on the treated [49, 50]. In addition, we included nesting of repeated measures within individual teina and teina within intervention group. We also included the conversational quality as a covariate. Conversational quality was measured by having the community researchers code the conversations using criteria of communication and conversation skills including listening, reflecting feelings, offering supportive comments, checking understanding, sharing experience, using vocal encouragement, and asking inviting questions [32]. The community researchers rated each criterion on a 3-point scale (very good, good, possible improvement) and an average of all the items was used to create a score of the conversational quality for the interaction. The analysis was separated by the two groups to better account for the implementation of the programmes during the COVID pandemic. In Aotearoa New Zealand, there were two significant lockdown periods. G1 was able to complete their programmes between the two lockdowns, whereas for G2 the programme was interrupted by the second lockdown period.

The second specific aim was addressed through the qualitative data. The interview transcripts, community researcher conversation comments, tuakana and teina focus groups, and community researcher final interviews/meetings document texts were coded using thematic analysis using criteria of recurrence, repetition, and forcefulness [51, 52]. Two researchers (one Māori, one Pākehā (people with settler heritage); both female) used Nvivo to firstly code the raw data using a thematic framework developed previously [18]. Secondly, the patterns of meaning across the data set in conversations were identified and (re)interpreted individually and collectively by the two researchers. This led to new themes being identified across the dataset. Together these processes led to decisions about the final themes. The themes were shared with participants, and they validated the themes without any recommended changes.

The third specific aim evaluated the cost-effectiveness of the programme using incremental cost effectiveness analysis (ICEA) [53, 54]. HRQOL was used as the as the primary outcome measure [55]. The incremental cost-effectiveness ratio (ICER) was converted to cost-per-QALY (Quality-Adjusted Life Year). Following WHO practice [54], cost-per-QALY was compared with GDP per capita to determine whether the programme was cost-effective or not. As a robustness check, cost-per-QALY was also compared with a measure of the value of a preventable life-year lost [56].

## Results

### Participants and participation

The average age of participants was 72.35 (SD = 8.23) and the sample was 76% female. Table 2 presents the demographic characteristics of the two programme groups at the pre-programme/baseline period. There were no significant differences in the demographic or outcome variables between the two groups at baseline.

**Table 2:** Teina at baseline

Variable	Category	G1	G2
Age		72.95 (7.96)	71.32 (8.69)
Biological Sex	Male	50	30
	Female	17	9
Relational Status	Married	24	10
	Widow	25	14
	Other	16	15
Who else lives in house	Partner	22	8
	Children	19	10
	Mokopuna	24	12
	Flatmate	2	2
	Others	6	6

Notes: Frequency or Mean (SD)

For the G1 group, there was a total of 71 teina at baseline. Of these teina, 57 completed the programme, with 14 withdrawals. For the G2 group there were a total of 42 at baseline and 29 completed the programme (13 withdrawals). The withdrawals occurred due to participants passing away (*n* = 10), becoming ill (*n* = 5), other commitments (*n* = 4) or unspecified (*n* = 8). Both groups averaged slightly over five of the six expected conversations. We completed a fidelity check of the conversations by independently coding the conversations for communication features covered in the orientation programme; these were completed by the local community researchers in most cases (for one provider in G2 was completed by a university researcher). The average ratings were 2.37 for both groups, demonstrating relatively high levels of communication between the tuakana-teina pairs. Table 3 displays information about the number of teina and conversation quality for each of the two groups.

**Table 3:** Conversations information and teina in the programme

Variable	G1	G2
Average Number of Teina per Tuakana	5.91	6.00
Average Number of Conversations per Pair	5.16 (SD = 1.54)	5.22 (SD = 1.31)
Conversation Quality	2.37 (SD = 0.50)	2.37 (SD = 0.38)
Number of Teina at Baseline	71	42
Number of Teina at Programme Completion	57	29
Withdrawals	14	13

### Mixed model outcomes

The first key aim was to have a positive impact on the hauora and mana motuhake outcomes for teina. Table 4 displays the means from baseline to post-programme and the effect, including the effect when the covariate (conversation quality) was included.

**Table 4:** Means of outcome variables for teina participating in the programme and programme effect

Variable	Baseline M(SD)	Group 1			Group 2		
		Post-Programme M(SD)	Effect (SE)	Effect with Conversation Quality Covariate (SE)	Post-Programme M(SD)	Effect (SE)	Effect with Conversation Quality Covariate (SE)
*Hauora Outcomes*							
HRQOL (100-point)	64.43(20.10)	68.62(17.48)	4.37(2.35)*	4.98(2.02)**	66.38(19.65)	1.98(3.51)	3.22(5.26)
Spiritual Wellbeing (100-point)	76.60(19.02)	74.45(20.32)	-2.11(2.87)	-1.62(2.30)	73.79(20.07)	-2.13(3.36)	-1.06(6.39)
Health Service Use and Knowledge (100-point)	70.44(21.10)	70.32(21.43)	-0.52(2.96)	-1.90(2.55)	68.87(21.04)	-1.62(4.21)	-0.31(5.43)
Loneliness (4-point)	1.61(0.64)	1.56(0.60)	-0.08(0.07)	-0.09(0.07)	1.70(0.45)	0.11(0.10)	0.08(0.10)
Needing more help with daily tasks (4-point)	1.92(0.82)	1.74(0.65)	-0.19(0.10)*	-0.19(0.11)*	1.90(0.82)	0.05(0.14)	0.07(0.14)
Needing more emotional support (4-point)	1.89(0.79)	1.75(0.64)	-0.14(0.10)	-0.13(0.07)*	1.71(0.46)	-0.18(0.11)	-0.32(0.28)
Cultural identity (5-point)	3.96(0.87)	4.13(0.85)	0.19(0.11)	0.14(0.12)	4.16(0.63)	0.04(0.10)	-0.01(0.10)
Knowledge of tikanga (4-point)	3.34(0.79)	3.17(0.75)	-0.14(0.09)	**-**	3.31(0.85)	-0.14(0.12)	-
*Mana Motuhake Outcomes*							
Elder Abuse (4 point)	0.15(0.27)	0.13 (0.22)	-0.02(0.03)	-0.03(0.03)	0.18(0.27)	0.03(0.05)	0.03(0.05)
Life satisfaction (10-point)	8.35(1.87)	9.04(1.08)	0.65(0.20)***	-	7.83(1.65)	-0.37(0.21)	-
Trouble Paying Bills (3-point)	1.39(0.75)	1.12(0.39)	-0.27(0.08)***	-0.29(0.07)***	1.17(0.38)	-0.22(0.09)**	-0.25(0.26)
Housing problems (4-point)	1.64(0.68)	1.48(0.69)	-0.13(0.06)**	-0.12(0.07)*	1.66(0.69)	0.05(0.11)	0.07(0.12)

*p < = 0.1; **p < = 0.05; *** p < = 0.01; effects not listed indicates model wouldn’t converge

Table 4 illustrates improvements from baseline to post-programme in most of the outcome variables and there were several significant effects for G1 including HRQOL, needing more help with daily tasks, life satisfaction, trouble paying bills and housing problems. Needing help with emotional problems was significant when considering the covariate of conversational quality. The only significant effect for G2 was for trouble playing bills. The smaller and non-significant effects for G2, when compared with the effects for G1, may have been primarily driven by the interruptions to the programme.

### Qualitative analysis: outcomes and resources received

This analysis addressed the second specific aim and supported the first specific aim. The findings are comprised of two main themes, which centre on how the tuakana-teina conversations supported the wellbeing of kaumātua with the greatest health and social needs. The first theme encapsulates the intangible dimensions of support experienced by teina in their conversations with tuakana. The second theme captures the tangible dimensions of support experienced by teina. Each theme has several subthemes. The discussion of each theme draws on comments by teina, tuakana, and community researchers. Table 5 displays the themes and an exemplar quote for each.

### Themes 1: intangible dimensions of support

This theme captured the intangible support related to the physical, social, emotional, spiritual, and cultural dimensions of wellbeing experienced by teina in their conversations with tuakana. Three support subthemes illustrated the impact on teina wellbeing: (1) Manaakitanga (care); (2) Wairuatanga (spiritual connection); and (3) Māoritanga (cultural connection).

#### Subtheme 1.1: Manaakitanga—Care

This subtheme concerned expressions or descriptions of tuakana support that helped teina in relation to their hauora tinana (physical wellbeing), hauora kare ā-roto (emotional wellbeing) and hauora hinengaro (mental well-being). Manaakitanga highlights the relational and cultural dynamics of support.

In respect of hauora tinana, the conversations allowed teina to talk about their physical health in a supported and culturally resonant way. For instance, teina commented, “I was able to talk to someone about my sickness and about my past history” (SurveysQual/Teina/Ref10) and “I could talk about my surgery for my head, for my tumor” (Teina3.3FG). A community researcher observed that because of the conversations, a teina “had a fantastic turn around with her wellbeing. Things have been rough for [the] teina and her whānau regarding her health. She is doing amazing health wise” (ConvObsCR4C6/10).

In respect of hauora kare ā-roto, satisfaction and comfort were recurring themes along with feelings of safety in sharing with tuakana. Specifically, teina described having taken the chance to “open up with someone you haven’t spoken to before, someone you don’t know, (…) it sort of comforts you [in] what they say back to you. They advise you and comfort you” (Teina3.1FG). The comfort within the relationship was part of emotional wellbeing in that teina “felt safe with [tuakana]. I’m not safe with a lot of people but [with] her I felt very, very safe” (Teina3.4FG).

Hauora hinengaro incorporates thoughts and feelings and was seen in the exchange of ideas and listening between tuakana and teina. A teina commented: that [her tuakana] was “always happy and cheerful. Someone who actually listened and not talked over you while you’re trying to talk. She listened and we swapped ideas. “We found a lot in common” (Tu-Te5.1FG/Ref/Te). Others said “I shared things with her that made me feel lighter” or talked of “sharing knowledge and weaving” ideas (Teina3.1FG).

#### Subtheme 1.2: Wairuatanga—spiritual connection

Wairuatanga refers to the spirit of connection between the tuakana and teina. This subtheme concerned how the relational dynamics helped teina realise spiritual wellbeing in their connections with tuakana and with their identity as kaumātua. Teina spoke of a burden being lifted and feeling “light” because of the conversations.

Teina comments included words and phrases such as “burden”, “buzz”, “light-hearted”, “peace”, “unloaded” and “weight was lifted”. A teina said, “I went away happy and light-hearted, the burden wasn’t heavy on my shoulders. It’s like a buzz when I left there. I don’t feel down, I feel nice and light” (Teina3.4/FG/Te). The comments show that teina spirits were lifted; the relationship, feeling safe, and the conversation helped to achieve this.

A second aspect of hauora wairuatanga was the change in teina spirits resulting from feeling encouraged by tuakana to talk about and reconsider how they saw themselves. As illustrated in the following comments, spiritual wellbeing included “opening up”, having “the courage to think” about themselves and other kaumātua (Teina3.4FG/Ref6) and “being empowered”. One teina highlighted that the conversation “was about me” and “the experience was fantastic” (Teina3.4FG/ref3). The phrase “that inner being makes you feel better” (see Table 5) relates directly to the essence of hauora wairuatanga.

Community researchers made similar observations, with one noting the value of safety in the conversations when she wrote, “Teina believes that this project has helped her immensely to bring this kōrero [talk about historical abuse] to light and eventually she will have the courage to tell her whānau soon” (ConvObsCR4C5/Ref4-5). Bringing the issue into the light enhanced hauora wairuatanga. The outcome was emancipation of spirit with the teina having “courage to tell her whānau soon.”

#### Subtheme 1.3: Māoritanga—Cultural connection

This subtheme involved teina in talking and processing issues with tuakana who were listening within Te Ao Māori. Cultural connection was supported strongly in the conversations, especially where the focus was mana motuhake, tūranga (roles) in later life, and tikanga Māori.

Teina described experiences that suggested being with and learning from other kaumātua contributed to cultural connectedness. For instance, a teina said, “Since I joined kaumātua/kuia (elder woman) my mana motuhake is kapai (good]. Spending time with kaumātua/kuia te korowai (wrap around support] makes me feel really empowered listening to their stories” (Surveys Qual/Teina/Ref1). One teina talked about being raised in a Pākehā lifestyle for 40 years in Australia. She returned home to “absorb Te Ao Māori” (ConvObsCR4C4) and the tuakana-teina conversations supported her reconnection.

Sharing with listening tuakana was a comforting and rewarding experience for many teina. A recurring theme was how teina developed their mana motuhake. Teina talked of “feeling more confident with being me” (Teina4.1FG/Te4) and “not about everyone else, and not about what I’m supposed to be doing” (Teina3.4FG). Another spoke of learning to ask questions of her tuakana (Teina3.2FG). In these comments, mana motuhake is seen in the growing confidence of teina to be and express themselves in their roles (e.g., of kuia) without being driven by implicit cultural expectations.

A significant aspect of cultural connection was connecting with whānau. Generally, teina identified the value of their conversations in gaining “knowledge about tools I can use with myself and my whānau” (SurveysQual/Teina/Ref6). Teina also talked of involving whānau in conversations about tikanga. One teina invited her daughter to join the conversation because it was about “collecting kai (food] and whose responsibility it is to ensure tikanga is practiced when gathering kai” (ConvObsCR4C5). Whānau members also noticed changes in the teina. One reported that her daughter said, “Gee, you fellas have calmed down now”, which the teina attributed to being “able to release the pressure” (Teina3.3FG/Te). Another commented on her son’s response: “’Mum, you sound good’, but I didn’t tell him where I’d been,…. I just said, ‘Oh, thank you, Son. I feel good, I feel good’” (Teina3.1FG/Te). These examples illustrate how the conversations helped to build whānau connectedness.

In summary, this theme highlights the value of intangible support provided by tuakana. Although addressed separately, the various dimensions of support enhanced the physical, mental, emotional, and spiritual wellbeing of teina, and their cultural and whānau connectedness. In this respect, the tuakana-teina conversations holistically supported the health and wellbeing needs of teina.

### Theme 2: tangible dimensions of support

This theme encompasses practical dimensions of support, including access to information and services for teina. The first subtheme focused on strengthening teina knowledge, and the second on meeting teina needs.

#### Subtheme 2.1: strengthening teina knowledge

This subtheme concerned strengthening teina knowledge about resources, services and information that could make a positive difference to their wellbeing. The tangible or practical dimensions of support discussed in the conversations included resource kete, fridge magnets, and booklets provided as part of the study, as well as external resources offered by tuakana and community researchers.

The overall sentiment was expressed by a teina who said “It was good to know that those services were there.… I didn’t realise [there was] so much resources for people to go and get help. Awesome” (Tu-Te5.1FG/Ref5-6). One provider distributed fridge magnets that listed community service contacts. Teina found this helpful for whānau “because the [phone] numbers are right there and for the traffic of whānau that come through my house, they all can see it” (Teina4.1FG/Ref1). Other teina also identified the value of knowing about available services and sharing them with whānau, as well as helping them to focus on their own wellbeing (see Table 5). Together, the comments show how the resources provided enhanced tuakana and teina knowledge about services and how this impacted on how they saw their own wellbeing.

Tuakana were a central source of advice and support that strengthened teina knowledge of services. Sometimes teina asked the tuakana about specific service information. One such request concerned “a $1000 annual finance grant” (ConvObsCR4C5/Ref7-8) available from a named agency. In another case, a teina reported that “My mokopuna [grandchildren] were surprised to know that I knew about the COVID tracking tool” (Teina4.1FG/Ref1/4).

#### Subtheme 2.2: meeting teina needs

This subtheme identified teina needs that were addressed during or because of the conversations. Such services were offered by tuakana or community researchers and used by teina to meet their needs.

Practical issues that arose in the conversations and were addressed as a result included a teina being “granted the $1000 which he used towards his treatment” (ConvObsCR4C5) and others “getting help with teeth” (Teina1.1FG/Ref1). In another example, a teina who was caring for her mother at home, asked the tuakana about, “Incontinence, because I didn’t have anyone to ask” (Teina3.2 FG/Ref1-3). Consequently, the teina was able to “ask that nurse in the office to put such-and-such tarau [pants] on mum today… instead of waiting for her to give me the push I was able to do it myself” (Teina3.2/FG/Te).

There were also important examples of tuakana support that impacted significantly on teina access to services and their wellbeing. In one situation, the tuakana and local coordinator-community researcher worked together to resolve a stressful situation (see Table 5). This example showed the value of the tuakana-teina relationship in identifying teina need, and the value of the tuakana-co-ordinator relationship in meeting that need. The relationship and subsequent action resulted in the teina getting access to appropriate support services to support him with his treatment at the hospital.

In some cases, information was offered that could help teina, but went unused. In one instance, a tuakana offered the teina support with giving up smoking. However, as much as she wanted to give up smoking, it gave the teina a sense of relief, and thus she did not want support. (ConvObsCR4C6). In another case involving a teina who was principal carer of her husband, the tuakana felt unable to provide support: “I feel like in some ways I could have given more for her. And the things that I did sort of suggest it was like, “Oh no, I don’t need that.” (Tuakana3.1FG). In both cases, teina refused the tangible support offered by tuakana, which can be viewed as teina exercising their mana motuhake.

Overall, this theme focused on how providing information strengthened teina knowledge and how the conversations helped teina to access services to meet their health needs. Although the examples showed this was achieved overall, sometimes teina chose not to act on potentially helpful information or services.

**Table 5:** Thematic Analysis Outcomes and Resources Provided

***Theme 1: Intangible Dimensions of Support***		
**Sub-themes**	**Illustrative Quotes**	
1. Manaakitanga—Care	I enjoyed my time with my [tuakana]. She was easy to engage with and I shared things with her that made me feel lighter. At my age we just hold on to it, but I have learnt that it made me feel good, once I shared my kōrero with her. (Teina4.1FG/Te4)	
2. Wairuatanga—Spiritual connection • Uplifted spirit • Empowerment	You come away really rangimārie (peaceful] and you’re at peace with yourself because you’ve been able to unload without feeling that you’ve unloaded. (Teina3.1/FG/Te) For me, being empowered it made me be better out there. I know I can get a bit hōhā (tiresome] other people get hōhā with me. It’s just that inner being makes you feel better. (Teina3.2FG)	
3. Māoritanga—Cultural connection • Mana motuhake • Whānau connectedness	This [these conversations] helped me get to that, to do my moko kauae (engraved markings on the chin], because it opened up the things I wanted to talk about, and I never ever thought of talking about it to anyone else because it wasn’t about me. It helped me look at my role as [a] kuia. (Teina3.4FG) This [is] a big step for the teina as she has previously felt uncomfortable to address her tangihanga (funeral] wishes with her children. She believes that because her children were not raised or immersed in Te Ao Māori, they wouldn’t give her a cultural service at the marae because they wouldn’t know how to. (ConvObsCR4C3/Ref1-2)	
***Theme 2: Tangible Dimensions of Support***		
*Sub-themes*	*Illustrative Quotes*	
1. Strengthening teina knowledge • Resources • Tuakana	The resource booklet was very helpful even though I thought I knew a lot of the services. Made me take more action of my wellbeing. (SurveysQual/Ref6) I [*tuakana*] said, “Oh, I’m getting my hearing aid.” He said, “Oh, they cost a lot.” I said, “No. I’m going to WINZ and I’m only paying back $20 a fortnight.” [he said]. “Eh, nobody told us that!” (Tu1.1 FG-A/Ref3-4)	
2. Meeting teina needs	The tuakana rung co-ordinator [community researcher] as she felt the teina was being mistreated in [named] hospital and he needed some support. He had an injury and upon arrival he was informed that he would have surgery the following day. He was also informed that he could not eat that night before surgery or have a big lunch as it’s not good to carry out the procedure. Five days later he still had no surgery, his pain increased, and he was hungry. Co-ordinator tended to the teina to advocate on his behalf. After six hours of engaging with local networks and Māori support workers from [the hospital], the co-ordinator felt comfortable to step back as the teina had good wrap around support. The outcome was, surgery had a confirmed date, his pain was assessed, and he could eat. (ConvObsCR4C6/Ref3)	

### Cost effectiveness analysis

The third aim was to examine the cost effectiveness of the programme. The comparison of unconditional means (in Table 4) shows a statistically significant effect for G1, but no significant effect for G2. However, as noted earlier, G2 received a programme that was interrupted by national lockdowns. We therefore estimated the cost effectiveness based on: (1) G1 only; and (2) both groups combined. The cost-effectiveness evaluation for G1 represents a ‘clean’ evaluation based on the intended and uninterrupted programme, while the effect for both groups combined is more conservative and consistent with the original analysis plan.

The total cost of the programme excluding evaluation costs was NZ$206,130 for both groups combined, and NZ$123,678 for G1. The cost of the status quo (no programme) was assumed to be zero. The coefficient (and standard error) for the cost effectiveness were taken from Table 4 (for G1) and was 2.98(1.78) for both groups combined. The ICERs were estimated 5000 times, using random draws from a normal distribution, based on the coefficient and standard error. The cost per unit increase in HRQOL for G1 was NZ$549 (95% C.I. $260, $1236), and for both groups combined was NZ$1033 (95% C.I. -$972, $3721). These estimates can be interpreted as the cost to raise one kaumātua HRQOL by one point (on a 0-100 scale). They can also be interpreted as the cost of 0.01 Quality Adjusted Life Years (QALYs), assuming that one QALY corresponds to an increase from the bottom to the top of the HRQOL scale (0-100). Scaling this estimate to the cost for one whole QALY results in an estimate for G1 of NZ$54,869 (95% C.I.$26,010, $123,590) and for both groups combined of NZ$103,330 (95% C.I. -$97,155, $372,075). Following WHO recommendations, this cost per QALY represents a cost-effective health intervention, as the cost per QALY averted is less than New Zealand GDP per capita (NZ$63,550 for the 2020 year) [57], regardless of whether we consider the point estimate for G1 alone or for both groups combined. It is also substantially below the conventional threshold of three times GDP per capita, below which programmes are considered cost-effective [62].

However, criticism has been levelled at GDP per capita as a threshold for assessing cost-effectiveness [58]. An alternative threshold is the value of a statistical life year lost (VSLYL), which has been estimated at NZ$130,295 (in 2008 NZ$) [56]. Again, assuming that this value is equivalent to a 100-point decrease in HRQOL, we again find that the programme is cost-effective, regardless of whether we look at G1 alone or both groups combined.

## Discussion

The aims of the study were to identify the impact on outcomes, resources provided and the cost effectiveness of the tuakana-teina programme. The analysis revealed improvements in HRQOL, help with daily tasks, life satisfaction, paying bills and housing problems. Qualitative results supported impacts of the programme on mana motuhake and hauora, through providing intangible and tangible resources. Cost-effectiveness analysis showed that the intervention is cost effective, with a cost per QALY substantially below than the conventional threshold of three times GDP per capita.

The current findings illustrate the positive outcomes of peer support and education with elders. Research demonstrates use of peer support with older populations in terms of issues such as increasing awareness of health issues [59, 60], palliative care [40, 61, 62], successful ageing [63], chronic condition self-management [64] and physical activity and fall-prevention [65–68]. The current study also further supports the previous research on the tuakana-teina programme to show positive impacts on hauora and mana motuhake outcomes for an elder Indigenous community [32]. The qualitative responses attribute positive impact of the tuakana-teina programme for participants’ hauora and mana motuhake as well, particularly in terms of mental, emotional, cultural, and spiritual wellbeing. More importantly, the qualitative findings document that the peer education process identified key health issues and provided information and access to resources that had not been identified previously. The tuakana-teina relationship provided a safe space to explore a variety of health issues for kaumātua with key needs.

Overall, the study illustrates that the tuakana-teina programme creates a culturally resonant social environment with important benefits to social connectedness and cultural renewal. The opportunity to connect culturally is significant given the history of devaluing and removing Te Ao Māori and Te Reo Māori for kaumātua when they were younger [69]. Further, the social connectedness is important as research finds greater social isolation for Māori relative to other New Zealanders [70], which is significant given its links to poor health [70, 71].

In addition to being effective, the programme was also cost-effective. The cost per QALY compares favourably with thresholds based on GDP per capita (and three times GDP per capita) and the value of a statistical life year lost. It also compares favourably with the original programme, being more cost-effective in comparison with that programme [32]. This may reflect a combination of improvements in the efficiency and efficacy of the programme. Identifying cost-effective interventions is a “moral imperative” [72] for public health care systems like that in New Zealand, particularly in a time of change and reorganisation of the health system, which began in 2022. Indeed, new health delivery options for Māori need a strong evidence base of cost-effective programmes to improve hauora Māori.

The programme demonstrates the importance of implementing the tuakana-teina programme through a participatory and co-design participatory process using an Indigenous-based framework [35]. The Indigenous participatory process ensures that the programme is grounded in kaupapa Māori and is kaumātua-led and provider-led. The implementation process enabled the new providers to adapt the programme to fit their local culture and take ownership of the implementation process [18]. Participatory processes are frequently used approached to work with Indigenous communities and to address health inequities [73]. This study provides further evidence of the benefit of the participatory approaches both for programme effectiveness and implementation effectiveness.

A further implication is the benefit of grounding the tuakana-teina programme in mātauranga Māori (knowledge) or kaumātua mana motuhake. This programme takes a strengths-based rather than the oft-used deficit approach. It aligns with mana motuhake and the programme utilised Māori culture itself for answers to health and social issues through the use of Māori epistemologies surrounding ageing [19]. The strong mana motuhake impacts in both the survey and qualitative research demonstrated kaumātua feelings of mana motuhake because of the tuakana-teina programme.

The study does have some limitations. First, we had one provider who was not able to use random selection of participants. Second, we used self-report measures without any direct outcome measures. Third, the study took place during the COVID pandemic and thus its implementation had some delays. It is a testament to the excellence and resilience of the providers that they were able to complete the programme despite the challenges created by the pandemic. Finally, the study was non-blinded, clustered, and open to contamination (i.e., participants sharing with others). These are generally unavoidable in a community trial. To mitigate these factors, we applied random selection in most communities and the staggered design was applied at a community level to limit sharing across the providers. However, we did not try to control contamination and blinding as to do so would be counter to the mana motuhake of kaumātua and them wanting to discuss the programme and know what was going on in the programme.

## Conclusions

This study resulted in a culturally resonant and effective tuakana-teina programme to assist kaumātua to talk about their health and social needs, as well as identify resources and services to assist them with these needs. The tuakana-teina programme is cost effective and has the potential to help Indigenous communities help address inequities in ageing. The positive outcomes in this project can be attributed to the focus on a participatory development process, an adaptable implementation process, and being kaumātua-led and focused on mana motuhake. The findings provide an innovative, strengths-based approach for kaupapa Māori community providers to address unmet needs for kaumātua. It illustrates how a few resources can result in strong outcomes when grounded in a culturally-resonant environment.

## Data Availability

The datasets used and/or analysed during the current study are available from the corresponding author on reasonable request.

## Abbreviations

G1: 1st group
G2: 2nd group
GDP: Gross domestic product
HRQOL: Health-related quality of life
ICEA: Incremental cost effectiveness analysis
ICER: Incremental cost-effectiveness ratio
QALY: Quality-adjusted life year

## Glossary

**Te Reo Māori**: **English Approximation**.
*Aotearoa*: New Zealand.
*aroha*: love, compassion, empathy.
*hauora*: wellbeing.
*hauora hinengaro*: mental wellbeing.
*hauora kare ā-roto*: emotional wellbeing.
*hauora tinana*: physical body wellbeing.
*hauora wairuatanga*: spiritual wellbeing.
*hōhā*: tiresome, boring, hassle.
*kai*: food, meal.
*kapai*: good.
*kare ā-roto*: emotions/emotional.
*kaumātua*: older people.
*kaumātua-kuia te korowai*: wrap around support for kaumātua and kuia.
*kaumātuatanga*: older people’s experiences and worldview.
*Kaupapa Māori*: research/services by Māori for Māori.
*kete*: basket.
*kōrero*: speak, talk, narrative.
*kuia*: older woman.
*mana*: status.
*mana motuhake*: identity, autonomy.
*manaakitanga*: Hospitality, generosity extended.
*Māori*: Indigenous people of Aotearoa New Zealand.
*Māoritanga*: relating to all aspects of Māori identity and culture.
*mātauranga*: Māori system of knowledge.
*mātāpono*: Principles.
*moko kauae*: Traditional engraved markings on the chin for women of rank.
*Pākehā*: New Zealander of settler heritage.
*pono*: honesty.
*rangimārie*: peace/fulness, quiet.
*tangihanga*: funeral in accordance with *tikanga Māori*.
*tarau*: pants.
*Te Ao Māori*: Māori worldview.
*Te Aka Whai Ora*: Māori Health Authority.
*Te Reo Māori*: the Māori language.
*teina*: junior to *tuakana* (the younger or less experienced).
*tika*: fair.
*tikanga*: cultural practices and protocols; cultural wellbeing.
*tūranga*: roles, stance.
*tuakana*: senior to *teina* (the older or more experienced).
*tuakana-teina*: older and younger, same sex sibling or cousin relationships; peer educator/peer learner in this study.
*wairua*: spirit/metaphysical.
*wairuatanga*: relating to the spiritual.
*whānau*: closely connected kin group/extended family.
*whanaungatanga*: social wellbeing.
